# Intranasal Location and Immunohistochemical Characterization of the Equine Olfactory Epithelium

**DOI:** 10.3389/fnana.2016.00097

**Published:** 2016-10-13

**Authors:** Alexandra Kupke, Sabine Wenisch, Klaus Failing, Christiane Herden

**Affiliations:** ^1^Institute of Veterinary Pathology, Faculty of Veterinary Medicine, Justus Liebig University GiessenGiessen, Germany; ^2^Institute of Virology, Philipps University MarburgMarburg, Germany; ^3^Small Animal Clinic c/o Institute of Veterinary Anatomy, Histology and Embryology, Department of Veterinary Clinical Sciences, Justus Liebig University GiessenGiessen, Germany; ^4^Unit for Biomathematics and Data Processing, Faculty of Veterinary Medicine, Justus Liebig University GiessenGiessen, Germany

**Keywords:** olfactory epithelium, subtypes, immunohistochemistry, horse, statistical analysis

## Abstract

The olfactory epithelium (OE) is the only body site where neurons contact directly the environment and are therefore exposed to a broad variation of substances and insults. It can serve as portal of entry for neurotropic viruses which spread via the olfactory pathway to the central nervous system. For horses, it has been proposed and concluded mainly from rodent studies that different viruses, e.g., Borna disease virus, equine herpesvirus 1 (EHV-1), hendra virus, influenza virus, rabies virus, vesicular stomatitis virus can use this route. However, little is yet known about cytoarchitecture, protein expression and the intranasal location of the equine OE. Revealing differences in cytoarchitecture or protein expression pattern in comparison to rodents, canines, or humans might help to explain varying susceptibility to certain intranasal virus infections. On the other hand, disclosing similarities especially between rodents and other species, e.g., horses would help to underscore transferability of rodent models. Analysis of the complete noses of five adult horses revealed that in the equine OE two epithelial subtypes with distinct marker expression exist, designated as **types a** and **b** which resemble those previously described in dogs. Detailed statistical analysis was carried out to confirm the results obtained on the descriptive level. The equine OE was predominantly located in caudodorsal areas of the nasal turbinates with a significant decline in rostroventral direction, especially for **type a**. Immunohistochemically, olfactory marker protein and doublecortin (DCX) expression was found in more cells of OE **type a**, whereas expression of proliferating cell nuclear antigen and tropomyosin receptor kinase A was present in more cells of **type b**. Accordingly, **type a** resembles the mature epithelium, in contrast to the more juvenile **type b**. Protein expression profile was comparable to canine and rodent OE but equine **types a** and **b** were located differently within the nose and revealed differences in its cytoarchitecture when compared to canine OE. Equine OE **type a** closely resembles rat OE. Whether the observed differences contribute to species-specific susceptibility to intranasal insults such as virus infections has to be further investigated.

## Introduction

The OE displays unique features of epithelia and nerve tissue in the body. It is the only site where neurons are directly exposed to the environment and therefore may be affected by a broad range of insults, including neurotropic virus infections. Moreover, disruption of smell has been detected in neurodegenerative diseases such as Alzheimer’s disease or Parkinson’s disease even before typical neurological signs occur (Doty, 2009; Benarroch, 2010; Holbrook et al., 2011). Many neurotropic viruses employ the olfactory pathway to enter the central nervous system (CNS) without recognition of the immune system, amongst them several herpesviruses, rabies virus, vesicular stomatitis virus (VSV), canine distemper virus, hendra and nipah virus as well as influenza virus A (reviewed by Reinacher et al., 1983; Mori et al., 2005; Salinas et al., 2010; Kalinke et al., 2011; McGavern and Kang, 2011; van Riel et al., 2015). In horses, a well-known example is the Borna disease virus (BoDV-1), order *Mononegavirales*, the etiologic agent of the classical Borna disease (Morales et al., 1988; Shankar et al., 1992, reviewed in Herden et al., 2013). To date, it is unknown whether specific features of the OE, e.g., location within the nose, maturity, turnover, cytoarchitecture, and protein expression profile contribute to successful intranasal infection in a certain species or even promote resistance via this route. Typically, experimental rodent studies were used to demonstrate successful intranasal virus infection (reviewed by Mori et al., 2005; Salinas et al., 2010; Kalinke et al., 2011; McGavern and Kang, 2011; van Riel et al., 2015), e.g., rat models for BoDV-1 (Morales et al., 1988; Shankar et al., 1992). Ideally, the model system should reflect the situation in the natural host but whether the cytoarchitecture, protein expression profile, maturity, turnover and location of the OE is conserved between rodent models and end hosts of natural infections, e.g., horses, often remains to be shown. Such a comparative approach has already been done for the morphological characterization of animal and human OE (Morrison and Costanzo, 1992; Deckner et al., 1993; Bock et al., 2009; Barrios et al., 2014a,b). So far species-specific differences have been discussed by Bock et al. (2009) and Barrios et al. (2014a) who compared the canine OE with rodent models. In dogs, two histomorphologically distinguishable types of the OE with opposing marker expressions have been described and interpreted as different maturity stages (Bock et al., 2009). This was, however, not confirmed by others (Barrios et al., 2014a). To date, knowledge on equine OE is very limited, it has only been characterized very roughly regarding its location in the caudal area of the ethmoturbinates near the cribriform plate (Kumar et al., 2000). The protein expression profile has so far only been reported for the equine vomeronasal organ (Garcia-Suarez et al., 1997).

In general, the OE is composed of three main groups of cells, namely the olfactory receptor neurons (ORNs), the sustentacular cells and the basal cells, which contain different cellular subtypes. A unique and striking feature of the OE is its life-long neurogenesis (Graziadei and Graziadei, 1979; Graziadei and Monti Graziadei, 1983) starting from the horizontal and globose basal cells which lie next to the basal lamina (Graziadei and Graziadei, 1979; Graziadei and Monti Graziadei, 1983; Farbman, 1990). The globose basal cells are thought to be the progenitors of the ORNs (Graziadei and Graziadei, 1979; Calof and Chikaraishi, 1989; Mackay-Sim and Kittel, 1991) and the sustentacular cells (Schwob et al., 1994; Chen et al., 2004). Due to their proliferative activity, globose basal cells can be visualized by BrdU (Carter et al., 2004) or [^3^H]-thymidine incorporation (Graziadei and Graziadei, 1979) and express proliferating cell nuclear antigen (PCNA) or Ki-67 (Miller et al., 2003; Carter et al., 2004; Bock et al., 2009; Holbrook et al., 2011). The horizontal basal cells possess features of multipotent stem cells and can induce regeneration after epithelial damage (Schwartz Levey et al., 1991; Holbrook et al., 1995; Carter et al., 2004; Murdoch and Roskams, 2007). They can be positive for several markers such as cytokeratins 5 and 6 (Schwob et al., 1995), the transcription factor Pax-6 (Davis and Reed, 1996), the epidermal growth factor receptor (EGFR) (Krishna et al., 1996), the intercellular adhesion molecule-1 (ICAM-1) and α_1_-, α_3_-, α_6_-, β_1_ und β_4_-integrin (Carter et al., 2004). Moreover, they express the high affinity nerve growth factor (NGF) receptor tropomyosin receptor kinase A (TrkA) and can therefore be stimulated by NGF (Feron et al., 2008). If the horizontal basal cells are progenitors of the globose basal cells or if they represent a different entity still needs to be investigated (Calof and Chikaraishi, 1989; Schwob, 2002; Carter et al., 2004; Murdoch and Roskams, 2007). The proliferating globose basal cells differentiate into juvenile neurons. Their nuclei are typically located in the middle third of the epithelium (Calof and Chikaraishi, 1989). Juvenile neurons express TrkB (Roskams et al., 1996; Feron et al., 2008), growth-associated phosphoprotein (GAP43) (Verhaagen et al., 1990) and doublecortin (DCX) (Carson et al., 2006; Murdoch and Roskams, 2007). The adult ORNs are bipolar neurons with an unbranched dendrite with dendritic knob and cilia that reaches the epithelial surface and an axon that is directed toward the basal lamina. The most specific marker for the ORNs is olfactory marker protein (OMP) (Hartman and Margolis, 1975; Farbman and Margolis, 1980). OMP expression is widely conserved from fish, amphibian up to mammals and man (Keller and Margolis, 1975; Rossler et al., 1998). Less specific markers are protein gene product 9.5 (PGP9.5), neuron-specific enolase NSE (Weiler and Benali, 2005) and TrkC (Roskams et al., 1996; Feron et al., 2008). The supporting or sustentacular cells are non-neuronal cells (Hempstead and Morgan, 1983) and are thought to be responsible for stability, detoxification, and phagocytosis (Weiler and Farbman, 1998). This cell type can be recognized by several markers such as cytokeratin 18 (Schwob et al., 1995), the so-called SUS-1 and SUS-4 (sustentacular cell-1 and -4) (Hempstead and Morgan, 1983; Goldstein and Schwob, 1996) or in case of carrying microvilli they express the marker 1A-6 (Carr et al., 1991).

A detailed characterization of specific morphological features of the equine OE, e.g., location within the nose, maturity, turnover, cytoarchitecture, and protein expression profile can be helpful to address many open questions regarding species-specific or species-conserved features in a comparative approach. Since this has been lacking for the horse, this was carried out for the equine OE and data were compared to rodent and canine data.

## Materials and Methods

### Animals

Noses of five adult horses (3, 5, 10, 20, and 21 years old, four geldings and one mare) were collected during routine necropsies at the Institute of Veterinary Pathology, Justus Liebig University Giessen. Determined by macroscopic and histopathologic examination, the horses did not suffer from any disease of the central nervous or respiratory system and all animals were euthanized for other medical reasons.

### Tissue Preparation and Histological Examination

**FIGURE 1:**
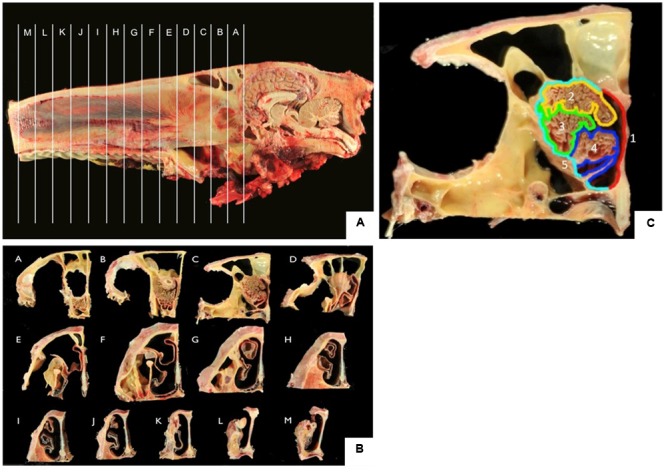
**(A,B)** Frontal sections A–M of the equine nose, beginning at the most caudal parts of the ethmoid bone (section A) and ending at the nares (section M); **(C)** five localizations for sampling including nasal septum (1, red), dorsal (2, yellow), mid (3, green), ventral (4, blue) part of the nasal turbinate, lining of the nasal cavity (5, turquoise).

After decapitation, heads were skinned and sawn along the longitudinal axis with a band saw. Tissue was fixed in 10% formalin. Of each nose, 13 frontal sections (sections A–M; **Figures 1A,B**) of 2 cm thickness were sawn, beginning with the transition between the olfactory bulb (A) and the cribriform plate and ending at the nares (M). The sections were subdivided into representative samples of five different localizations (localizations 1–5; **Figure 1C**) of the nasal septum (1), the dorsal (2), the mid (3), and the ventral part (4) of the turbinates as well as the outer lining of the nasal cavity (5). Each area was decalcified in EDTA at 37°C for 8–14 days, rinsed in water overnight and routinely embedded in paraffin. Paraffin sections (4 μm thickness) were used for H&E-staining in an autostainer (Microm, HMS 740) or for immunohistochemistry.

### Semiquantitative Analysis of the OE and the Subtypes

By light microscopy (10x magnification), the percentage of the OE in relation to the whole epithelial tissue visible on the slide was determined in 20%-steps (range from 0 to 100%). This was carried out for each of the five localizations of the 13 cross sections of the equine nose. Afterward, the percentage of the whole OE was subdivided, corresponding to the two morphologically distinguishable types of the OE. The subtypes are further referred to as **types a** and **b.**

### Immunohistochemistry

The sections were further used for immunohistochemistry (**Table 1**). All steps were performed at room temperature. Slides were deparaffinized in xylol (15 min) and a descending series of ethanol. Endogenous peroxidase was blocked by incubation in pure methanol containing 0.05% H_2_O_2_ for 30 min. Primary antibodies were incubated over night at 4°C in a humid chamber. After incubation of secondary antibodies, detection systems and several washing steps (**Tables 2** and **3**), the slides were incubated in DAB (3,3-diaminobenzidine-tetrahydrochloride, Sigma-Aldrich) with H_2_O_2_ in 0.1 M imidazole, pH 7.1, for 3 min to visualize specific antigen-antibody binding. Slides were counterstained with Papanicolaou stain. For the goat- and rabbit-antibodies non-immune sera of the corresponding species served as negative control and a mouse monoclonal antibody against chicken lymphocytes when using mouse monoclonal antibodies (Hirschberger, 1987).

**Table 1:** Primary antibodies, blocking, and detection system for immunohistochemistry.

Primary antibody	Clonality	Dilution	Blocking
–	–	–	–
Supplier	Specitivity	Pretreatment	Detection system
Goat anti-olfactory
marker protein (OMP)	Polyclonal	1:3000 in TBS + 1% BSA	20% HS, 20 min
–	–	–	–
Wako Chemicals USA, Inc., Richmond, VA, USA	Rodent OMP	Citrate (pH 6,0), 23 min	ABC

Goat anti-Doublecortin [DCX (C-18)]	Polyclonal	1:250 in TBS + 1% BSA	20% HS + 0.3% Triton^®^ X-100, 60 min
–	–	–	–
Santa Cruz Biotechnology, Inc., Santa Cruz, CA, USA	Epitope at the C-terminus of human DCX	Citrate buffe (pH 6,0), 23 min	Streptavidin

Mouse anti-Proliferating cell nuclear antigen (PCNA)	Monoclonal	1:100 in TBS + 1% BSA	10% RS, 10 min
–	–	–	–
Dako	Clone PC 10	Citrate buffer (pH 4,0), 23 min	Mouse PAP

Rabbit anti-tropomyosin receptor kinase A [TrkA (763)]7	Polyclonal	1:100 in TBS + 20% PS	20% PS, 15 min
–	–	–	–
Santa Cruz	Epitope near the C-terminus of human TrkA	None	ABC

*TBS, tris buffered saline; BSA, bovine serum albumin; HS, horse serum; PAP, peroxidase anti-peroxidase method; PS, porcine serum; ABC, avidin–biotin complex method.*

**Table 2:** Secondary antibodies used in immunohistochemistry.

Secondary antibody	Supplier	Dilution
Goat anti-rabbit IgG (H+L), biotinylated	Vector Laboratories, Inc., Burlingame, CA, USA	9 μl in 1000 μl TBS + 20% PS
Horse anti-goat IgG (H+L), biotinylated	Vector Laboratories	9 μl in 1000 μl TBS + 1% BSA
Rat anti-mouse IgG (H+L)	Jackson ImmunoResearch Laboratories, Inc., West Grove, PA, USA	1:100 in TBS + 1% BSA

*TBS, tris buffered saline; PS, porcine serum; BSA, bovine serum albumin.*

**Table 3:** Detection systems used in immunohistochemistry.

Detection system	Supplier	Dilution
Avidin–biotin complex (Vectastain^®^ ABC Kit Peroxidase Standard)	Vector Laboratories	9 μl Avidin (A) + 9 μl Biotin (B) in 1000 μl TBS
Mouse peroxidase anti-peroxidase (PAP)	Jackson ImmunoResearch	1:500 in TBS
Streptavidin (HRPO Conjugate)	Invitrogen	1:500 in TBS

*TBS, tris buffered saline.*

### Semiquantitative Analysis of the Immunohistochemistry

According to the presence of OE analyzed by H&E-staining, percentage of number of cells expressing the respective marker was assessed applying the following scoring system: (0) no positive cells; (1) 1–30% of cells express the respective marker; (2) 30–70% of cells express the respective marker; (3) more than 70% of cells express the respective marker.

### Statistical Analysis

The statistical analysis was done by means of the statistical software packages BMDP (Dixon, 1993) and StatXact (Cytel Studio, 2010) to confirm the tendencies in the differing percentages of OE by detailed statistical analyses. On the one hand the design of the study was multi-factorial with repeated measures on the same individual. On the other hand the statistical scale level of the observed variables was an ordinal one. Neither the conditions for the application for parametric nor for non-parametric statistical procedures were given exactly. Therefore the analysis was done in different steps in an exploratory manner.

(1) Percentage of the OE:

As a first step (I) a two-way ANOVA with repeated measures for section and localization was performed using the program BMDP2V to determine the relationship between percentages of OE and the respective section or localization and if an interaction between both factors was present. Moreover, based on the differences between the sections within the localizations, as a second step (II) the exact Friedman test served to test for a possible interaction between localization and section in a non-parametric manner. Two additional steps compared the localizations section-wise (III) and the sections localization-wise (IV) to test the main effects of these factors non-parametrically (Supplementary Table S1).

(2) Subtypes of the OE:

As a first step (I), a three-way ANOVA with repeated measures for section, localizations and subtype of the OE was carried out with the same program to test relationship between amounts of the subtypes **a** and **b** of the OE with the respective section or localization. Furthermore, data were checked for dual or triple interactions of all components. As a second approach, an exact Friedman test evaluated interactions between section and localization for OE **type a** or **b** (II). In two additional steps, the localizations were compared section-wise (III) and vice versa (IV). The same steps (II–IV) were performed for the sections and the subtypes of the epithelium split up for the localizations as well as for the subtypes and the localizations split up for the sections. If no interaction was verifiable, as a last step (V), the arithmetic means were compared for a global evaluation of the percentages of the OE.

In all cases the Bonferroni–Holm method served for α-adjustment of the multiple exact Friedman tests (Supplementary Table S1).

(3) Protein expression profile of the OE:

For the immunohistochemical data a three-factorial ANOVA with repeated measures of section, localization and marker was performed (step I). Furthermore, marker expression and type of OE were analyzed by the Spearman’s rank correlation (*r*_s_) using the program BMDP3D (step II) (Supplementary Table S1).

Unless otherwise stated, all results were accepted as statistically significant when *p* ≤ 0.05.

## Results

### Localization of the OE within the Equine Nose

All tissue samples were taken from representative regions of the five localizations comprising the nasal septum (area 1) as well as the dorsal, mid and ventral parts of the nasal turbinates (areas 2–4, respectively) and the outer lining of the nasal cavity (area 5) of the cross sections A–M (**Figure 1**).

The OE was histologically detectable in 3 of 5 horses in sections A–D, whereas, in the remaining two horses, it was only detectable in sections A–C. This did not correlate with the age of the horses. Sections E–M were covered exclusively by respiratory epithelium in all horses, whereas in rostral regions – near the nares – cuboid and stratified cornified epithelium was observed.

### Semiquantitative Analysis of the OE and Statistical Analyses

**FIGURE 2:**
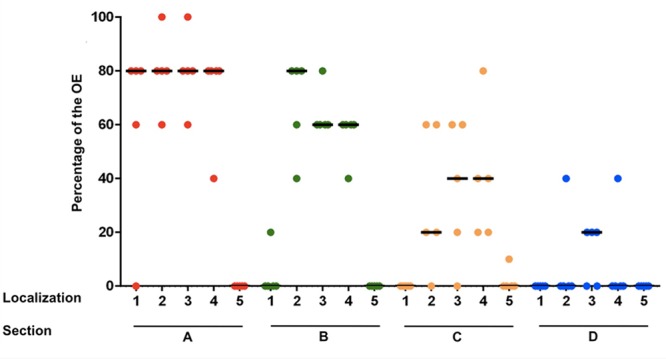
**Percentage of the olfactory epithelium (OE) of the whole epithelium in five equine noses for all sections containing OE **(A–D)**, split up for localizations 1–5; (1) nasal septum, (2) dorsal, (3) mid, (4) ventral part of the nasal turbinates, (5) lining of the nasal cavity; bar: median**.

In section A, localization 1 (nasal septum), in 4 of 5 horses 60–80% of the epithelium was classified histologically as OE. In section B the OE was only detectable in one horse with a percentage of about 20% in the nasal septum. In sections C and D no OE was seen in this localization. Thus, a strong decrease of the OE in rostral direction was observed for the nasal septum. In the dorsal part of the nasal turbinates (localization 2) a reduction of the OE was also found from caudal to rostral. In section A between 60 and 100% of the epithelium was recognized as OE and in section B between 40 and 80%. In 4 of 5 horses OE was still visible in section C with a percentage between 20 and 60% and in section D OE was only detectable in one horse with a percentage of about 40%. Presence of OE was comparable in the mid and ventral parts of the nasal turbinates (localizations 3 and 4, respectively). For localization 3 the percentage decreased from between 60 and 100% in section A, to 60–80% in section B and 20–60% in section C. In section C OE was found only in four out of five noses. In section D OE was present in 3/5 horses at a share of about 20%. In the ventral parts of the turbinates (localization 4) the amount of OE also declined in rostral direction. Whereas, in section A between 40 and 80% of the epithelium was identified as OE, only between 40 and 60% were found in section B. The amount finally decreased to 20 up to 80% in section C. In section D OE was only detectable in one horse with a percentage of about 40%. In localization 5 (lining of the nasal cavity) OE was only found in one horse but in low amounts (10% in section C at the transition to the nasal turbinates). Altogether, the highest amount of OE was present in the caudodorsal parts of the nasal turbinates and it declined continuously and significantly in rostral direction (**Figure 2**; Supplementary Table S2A).

As a first step (I) a two-way ANOVA based on the arithmetic mean of the percentages of OE (Supplementary Table S2A) was performed. This was done to examine exploratively the different percentages of OE in sections and localizations. By this global comparison differences between sections as well as differences between localizations were highly significant (*p* < 0.0001). Moreover, a significant interaction between section and localization was found, thus, different percentages of the OE in the localizations were dependant on the respective sections (*p* < 0.0001). Hence, the amount of the OE of the nasal septum and the nasal turbinates decreased from caudal to rostral in sections A–D, whereas in sections A and B a decrease from dorsal to ventral was observed additionally. For the lining of the nasal cavity, OE was detectable only sporadically at the transitional zones to the nasal turbinates.

These results were confirmed by the non-parametric exact Friedman test, including the Bonferroni–Holm method for α-adjustment in order to compare the differences between the sections. First (II, Supplementary Table S3A), the differences of sections B–D were taken and compared to section A, which served as reference. Significant interactions between section and localization were found for sections C and D, while for section B the results were not significantly different because of its strong similarity to section A. Afterward (III, Supplementary Table S3A), it was confirmed that the differences between the localizations were significant if compared section-wise for sections A–C. Vice versa (IV, Supplementary Table S3A), it was found that the different percentages of OE between the sections were statistically significant when split up for the localizations.

### Morphology of the Subtypes of the OE

**FIGURE 3:**
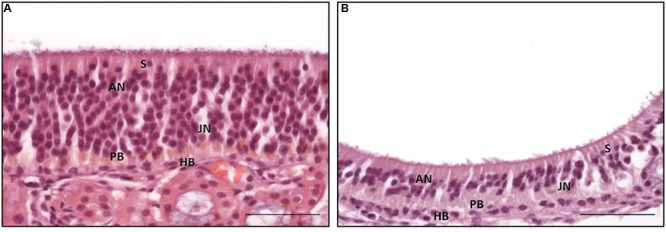
**Distinct subtypes of the olfactory epithelium; **(A)** OE type a with numerous adult olfactory neurons with euchromatic nuclei and distinct nucleoli in the middle third of the epithelium reaching toward the luminal surface, **(B)** OE type b with a reduced number of adult olfactory neurons forming a nucleus-free zone near the luminal surface; S: sustentacular cells, AN: adult neurons, JN: juvenile neurons, PB: proliferating basal cells, HB: horizontal basal cells; H&E staining; scale bar: 50 μm**.

The general structure of the OE and the cell morphology were comparable to those described for other mammalians (Graziadei and Graziadei, 1979; Morrison and Costanzo, 1992; Murdoch and Roskams, 2007). Interestingly, two morphologically distinct subtypes of the OE, designated **types a** and **b**, were found (**Figures 3A,B**) and resembled, in parts, the subtypes described for the dog (Bock et al., 2009). As in the dog, a patchy distribution of the subtypes, both existing side by side, was observed (Bock et al., 2009).

**Type a** was characterized by often more than 15 layers of nuclei (**Figure 3A**). The apically orientated nuclei formed an irregular surface with nuclei reaching close to the luminal pole of the epithelium. Moreover, the surface of the epithelium was characterized by irregularly distributed cilia. However, the general composition of the OE appeared unstructured. Mature ORNs were the dominating cell population and showed a round, euchromatic nuclei with one or two distinct nucleoli (**Figure 3A**). The immature ORNs were located more basally and contained an oval nucleus with more heterochromatin and less distinct nucleoli (**Figure 3A**). The number of juvenile ORNs was lower in comparison to the mature ORNs. OE **type b** was characterized by a straight border of the apically located nuclei, forming a broad nearly nucleus-free zone toward the luminal surface with only few nuclei of sustentacular cells (**Figure 3B**). The luminal surface had a more smooth appearance in comparison to **type a**. The epithelium was mostly built of less than 10 layers of nuclei, in some parts only five layers were visible. The general structure appeared more sorted but also loosened, due to less cell rows and a reduced number of adult ORNs, whereas the number of juvenile ORNs appeared equal in both types. Areas with a clear predominance of the juvenile ORNs as proposed by Bock et al. (2009) for the canine OE, were missing in all five examined horses.

### Semiquantitative Analysis of Distribution of the Subtypes of the OE and Statistical Analyses

**FIGURE 4:**
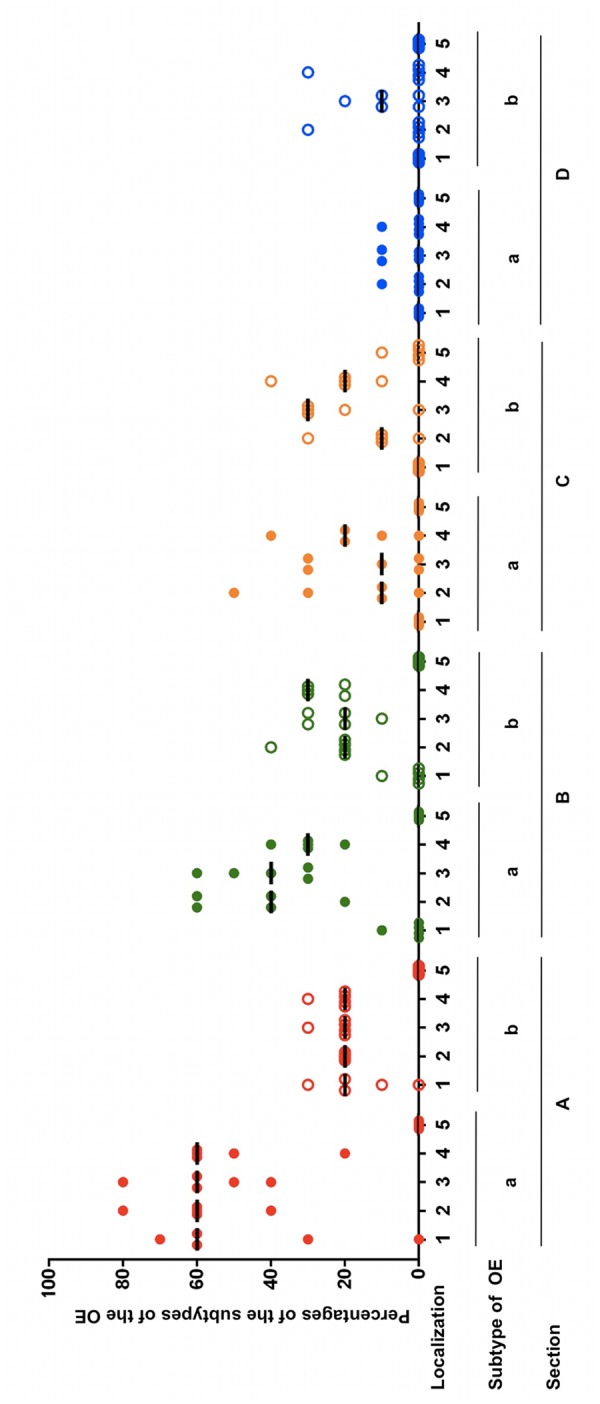
**Percentages of the subtypes a and b of the OE in five equine noses for all sections containing OE **(A–D)**, split up for localizations 1–5; (1) nasal septum, (2) dorsal, (3) mid, (4) ventral part of the nasal turbinates, (5) lining of the nasal cavity; bar: median**.

Within almost all frontal sections of the equine nose, both types of the OE were detectable side by side but differences in the percentages of the subtypes **a** and **b** were clearly visible (**Figure 4**; Supplementary Table S2B). For the most caudal section A, in all localizations the proportion of **type a** was higher than of **type b**. In localization 1 (nasal septum) of section A the percentage of OE **type a** ranged between 30 and 70% in four out of five horses, while **type b** was present in about 10–30%. One horse did not have any OE in this localization. In the dorsal part of the nasal turbinates (localization 2) the percentages were between 40 and 80% for **type a** and 20% for **type b**. In the mid and ventral parts (localizations 3 and 4), the amount ranged between 40 and 80% or 20 and 60%, respectively, for OE **type a** and for both localizations between 20 and 30% for **type b**. In section A no OE was seen in localization 5 (outer lining of the nasal cavity). In the more rostrally located section B only little OE was detectable on the nasal septum of one horse and only in the transitional area to the nasal turbinates. **Types a** and **b** shared equal amounts of about 10%. In the region of the nasal turbinates similar tendencies were observed as in section A, though less apparently. From the dorsal to the ventral part the percentages of **subtype a** ranged from 20 to 60%, 30–60% and 20–40%, respectively. The amount of epithelium **type b** ranged between 20 and 40% in the dorsal, 10 and 30% in the mid and 20 and 30% in the ventral part. No OE was detected in the lining of the nasal cavity (localization 5). In section C no OE was present on the nasal septum. In comparison to sections A and B the nasal turbinates revealed a different distribution pattern. In the dorsal part between 10 and 50% of the OE was identified as **type a** in four out of five horses, in the mid part between 10 and 30% in three out of five horses and in the ventral part between 10 and 40% in four out of five horses. The percentages of **type b** were 10–30% in the dorsal part, 20–30% in the mid part in four out of five noses and 10–40% in the ventral part. In one location about 10% OE **type b** could be seen at the outer lining of the nasal cavity near the nasal turbinates. As in section C, in section D no OE was found in localization 1 (nasal septum). In the nasal turbinates only small areas were covered with OE. In the dorsal and ventral part of the nasal turbinates (areas 2 and 4, respectively) OE was present only in one horse at about 10% of **type a**, while in the mid part of the nasal turbinates this was the case for 2/5 noses. In areas 2–4 **subtype b** predominated. In the dorsal and ventral part in one horse 30% **type b** was found, whereas in the mid part in 3/5 noses presence of OE **type b** ranged between 10 and 20%.

Statistically (I) a three-way ANOVA with repeated measures with respect to section, localization and type of epithelium was carried out first. This was done to assess exploratively which subtype of olfactory epithelium (OE) dominated in which section and in which localization. By this global comparison, highly significant differences between the percentages of the total OE were present for either for the sections and for the localizations (*p* < 0.0001) but not for **subtypes a and b** (*p* = 0.0697). However, the pairwise interaction between section and localization (*p* < 0.0001), between section and subtype of epithelium (*p* < 0.0001) as well as between localization and subtype of the OE (*p* = 0.0091) were statistically highly significant and this was also found for the triple interaction between section, localization and type of OE (*p* < 0.0001). Thus, the differences in the percentages of the distinct epithelial subtypes depended on the combination of section and localization.

The non-parametric exact Friedman test, including the Bonferroni–Holm method for α-adjustment was used to confirm the differences between the sections. First, the presence of a general interaction between section and localization for the epithelial **subtypes a** or **b** was determined. For this purpose, differences of the sections were compared to section A as reference (II, Supplementary Table S3B). For the OE **type a** significant differences were found for sections C and D but not for section B which closely resembled section A. Furthermore, the differences in the percentages of **OE type a** within the localizations were statistically significant when compared section-wise as confirmed for sections A–C (III, Supplementary Table S3B). Correspondingly, the differences in the amount of the OE within the sections were statistically significant when compared localization-wise (IV, Supplementary Table S3B). Because of lack of interaction between section and localization for **type b**, the arithmetic means of the localizations were examined globally for the sections and vice versa. Hence, percentages of **OE type b** were statistically significant different in a global statistical comparison (V, Supplementary Table S3B).

Moreover, statistically significant interactions between subtype of OE and section were found for all three areas of the nasal turbinates (localizations 2–4, respectively) (II, Supplementary Table S3C). For localization 1 the differences were only statistically significant when both subtypes were evaluated together (V, Supplementary Table S3C). For localizations 2–4 significantly different percentages between sections were only detected for epithelium **type a** (IV, Supplementary Table S3C).

Finally, statistically significant interactions between the subtypes of the OE and the localizations were confirmed for sections A and B (II, Supplementary Table S3D). Significant differences between the localizations were found for OE **type a** as well as for **type b** for the cross sections A and B (IV, Supplementary Table S3D). Additionally, for section C statistically significant differences between the localizations were confirmed for the mean of the subtypes.

In summary, in equines, the OE **type a** was predominantly found in the two most caudally located cross sections of the nose, sections A and B with a decrease from caudodorsal to rostroventral. For sections C and D an increase of OE **type b** was observed which was the dominating type in the areas of the nasal turbinates but the amount of OE was overall reduced in this area.

### Immunohistochemistry and Statistical Analyses

The cytoarchitecture of the equine OE was analyzed for the presence of four important cell types. Adult ORNs were detected by expression of OMP, juvenile ORNs by DCX positivity, globose basal cells by PCNA expression and horizontal basal cells by TrkA positivity. Immunohistochemistry was performed for all areas where OE was present in all five horses.

#### Olfactory Marker Protein

**FIGURE 5:**
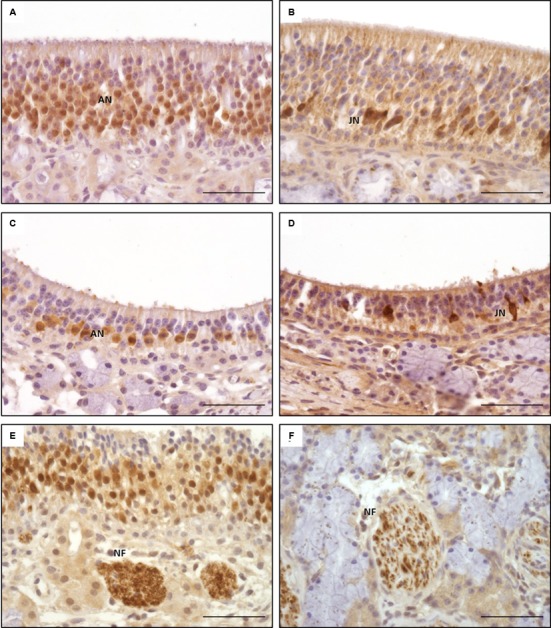
**Immunohistological detection of olfactory marker protein (OMP; **A,C,E**) and doublecortin (DCX; **B,D,F**); **(A,B)** olfactory epithelium type a; **(C,D)** olfactory epithelium type b; **(E,F)** nerve fibers in lamina propria; OMP staining of nuclei of adult olfactory neurons dominating in OE type a **(A)**, pronounced staining of cytoplasm in type b **(C)**; DCX staining of nuclei and cytoplasm of juvenile olfactory neurons in both types of OE **(B,D)**; ANs, adult neurons; JNs, juvenile neurons; NFs, nerve fibers; scale bar: 50 μm**.

Olfactory marker protein expression was found as strong dark-brown granular or diffuse staining of nuclei located in the mid third of the OE, in some localizations also reaching more basally located nuclei (**Figure 5A**). In some neurons, especially in neurons of OE **type b**, OMP was also detected diffusely in the cytoplasm resulting in a distinct staining of the dendrite up to the surface of the OE (**Figures 5A,C**) and a patchy or diffuse staining of the nerve fibers within the lamina propria underneath the OE (**Figure 5E**). Altogether, OMP served as reliable marker to detect adult ORNs in both subtypes of the equine OE with a more pronounced cytoplasmic expression in neurons of the OE **type b**. In sections A and B, either 30–70% (score 2) or even more than 70% (score 3) OMP positive neurons were found in areas containing OE (**Figure 7A**). Lower numbers of OMP positive neurons were found in the more rostrally located areas of sections C and D. OMP expression decreased remarkably in caudo-rostral direction with most prominent staining of the ORNs in localizations 2–4 (dorsal, mid and ventral parts of the nasal turbinates) of sections A and B.

For the nasal septum (localization 1) of section A between 30 and 70% OMP positive cells were found (score 2) but segments with no or more than 70% positive cells (score 3) were also present. For section B OMP expression was reduced to between 0 and 30% positive cells (score 1). Maximally, between 30 and 70% (score 2) of the neurons expressed OMP in this area (**Figure 7A**). In all three areas of the nasal turbinates (localizations 2–4) the score for section A was 2 or 3 indicating high numbers of OMP positive cells. A slight decrease in the number of OMP positive neurons was observed for localizations 2 and 4 within section B, but was constant for localization 3 when compared to section A (scores 2 and 3). Amount of OMP-positive cells decreased further in section C with a mean score between 1 and 2 in the localizations 2–4. Especially in localizations 2 and 3 OMP expression was variable, resulting in regions with extensive expression and regions with literally no staining for OMP. For section D only few epithelial segments revealed OMP expressing cells mostly as score 1 but some regions revealed between 30 and 70% (score 2) positive neurons (**Figure 7A**). Regarding the lining of the nasal cavity (localization 5) only few OMP positive neurons were detected in section C (score 1) for all horses examined (**Figure 7A**).

#### Doublecortin

Intracellular DCX expression was comparable to OMP staining pattern in mature ORNs and was characterized by a granular or diffuse staining of the nuclei. These nuclei were located more basally than the ones of the adult ORNs (**Figures 5B,D**). Additionally, DCX was found in the cytoplasm, including the dendrite but without reaching the luminal surface. Even though this cytoplasmic staining was not found in all areas examined it could be present in both subtypes of the OE (**Figures 5B,D**). DCX positive cells and nuclei were most often oval-shaped in contrast to the mature ORNs which typically display a round nucleus. DCX positive nerve fibers were found in the lamina propria, similar to the OMP positive fibers albeit staining was weaker (**Figure 5F**). As for OMP expressing neurons, number of DCX positive cells decreased in rostral direction, and most DCX positive cells were found on the nasal turbinates of sections A–C (**Figure 7B**).

For localization 1 within section A the mean score of DCX positive cells ranged between 1 and 2 in all horses but was reduced in section B as found for OMP positive neurons. No DCX specific staining was detected in sections C and D (**Figure 7B**) in this area. For the dorsal parts of the nasal turbinates (localization 2) the mean of positive cells reached score 2 while in the mid and ventral parts (localizations 3 and 4) number of DCX positive cells correspond to scores 1 or 2 in section A. In contrast to the tendencies seen for OMP, in section B more cells expressed DCX in localizations 2 and 3 than in section A with a mean score of about 2. Less DCX positive neurons were found in the ventral parts of the nasal turbinates (localization 4), score between 1 and 2 of section B. In section C the mean amount of DCX positive cells was reduced in localizations 2 and 3. Interestingly, regions with no staining for DCX (score 0) alternate with parts with more than 70% positive cells (score 3). In localization 4 number of DCX expressing neurons correspond to score 2 indicating an increase of DCX positive cells. In section D, number of DCX positive neurons decreased in all three areas of the nasal turbinates. As for OMP, in localization 5 of section C only few juvenile DCX positive neurons were found (**Figure 7B**).

#### Proliferating Cell Nuclear Antigen

**FIGURE 6:**
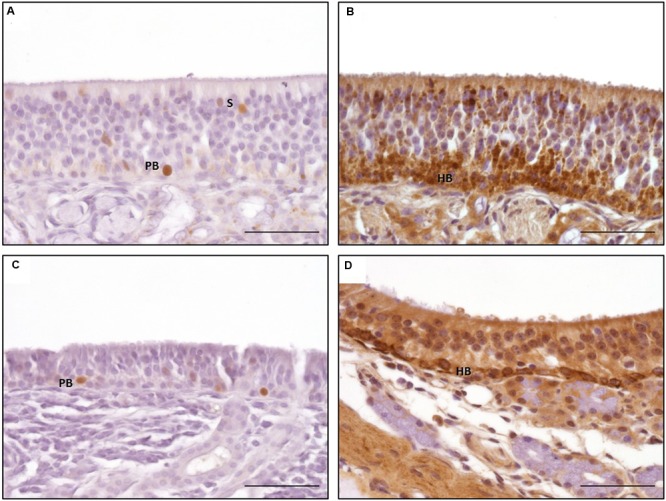
**Immunohistological detection of proliferating cell nuclear antigen (PCNA; **A,C**) and tropomyosin receptor kinase A (TrkA; **B,D**); a, b: olfactory epithelium type a; **(C,D)** olfactory epithelium type b; PCNA staining of nuclei of proliferating basal cell in both types of OE **(A,C)**; granular TrkA staining of the horizontal basal cells and sustentacular cells in OE type a **(B)** and ring-like staining of the plasma membrane of the horizontal basal cells in OE type b **(D)**; PB: proliferating basal cells; HB: horizontal basal cells; scale bar: 50 μm**.

**FIGURE 7:**
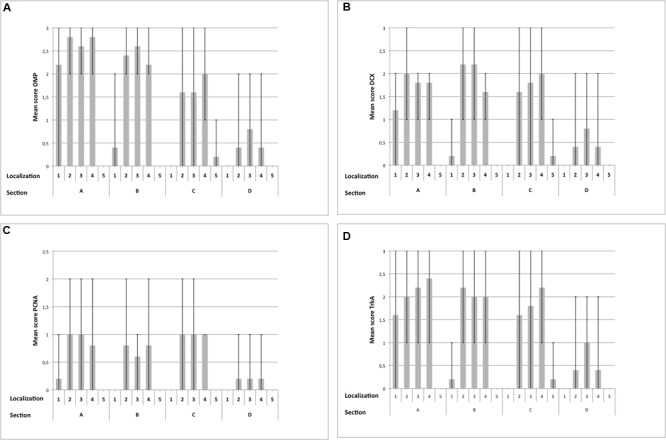
**Immunohistological detection of OMP **(A)**, DCX **(B)**, PCNA **(C)** and TrkA **(D)** for sections containing OE **(A–D)**, split up for localizations 1–5; (1) nasal septum, (2) dorsal, (3) mid, (4) ventral part of the nasal turbinates, (5) lining of the nasal cavity; bar: arithmetic mean; error bars: minimum/maximum value**.

Proliferating cell nuclear antigen expression was found as spotted or diffuse staining of the nuclei which were located near the basement membrane. No immunostaining was seen in the cytoplasm. In contrast to OMP and DCX expression, only scattered regions of the OE harbored cells positive for PCNA. Both subtypes of the OE contained PCNA positive cells without any difference in the staining pattern (**Figures 6A,C**). Additionally, a comparable immunostaining was visible in some nuclei of the supporting cells and in some glandular cells underneath the epithelium. Altogether, a score of up to 2 (30 and 70% positive proliferating basal cells) was found in few areas of the OE (**Figure 7C**). Only few PCNA positive cells were found in the nasal septum (localization 1) and only in section A with a maximum of score 1. Regarding the three areas of the nasal turbinates (localizations 2–4) PCNA expression was comparable within one section. For section A, typically not more than 30% PCNA positive cells were detected (score 1). A slight reduction was noticed for section B, but still between 0 and 30% cells expressed PCNA. In contrast, the number of PCNA positive cells increased slightly in section C (mean score 1) and in section D not more than 30% cells expressed PCNA (score 1) at maximum. Expression of PCNA was absent in localization 5 (**Figure 7C**) in all sections.

#### Tropomyosin Receptor Kinase A

Tropomyosin receptor kinase A expression was found in horizontal progenitor cells of the OE. Sustentacular cells and glandular cells in the lamina propria could be TrkA positive as well. The staining pattern comprised a granular cytoplasmatic or a circle-shaped labeling of the cellular membrane, in some cells a nuclear TrkA expression was seen as granular or diffuse staining. The apical cytoplasm of the supporting cells could also be TrkA positive (**Figures 6B,D**). TrkA expression was present in **subtypes a** and **b** of the OE even though not all areas were stained to the same extent. The ring-like staining of the horizontal basal cells was more prominent in cells of OE **type b** (**Figure 6B**). In general, TrkA positive cells were found variably within one histological slide or within one cross section but amount of TrkA positive cells did not decrease that obviously in rostral direction as observed for OMP and DCX expression. Number of TrkA positive cells decreased first in section D.

For localization 1 of section A areas with score 0 alternate with segments with more than 70% TrkA positive cells (score 3). In section B only few cells expressed TrkA (**Figure 7D**) in this region. In all three areas of the nasal turbinates of section A TrkA expression was patchy with areas with few TrkA positive cells (score 1) but also with segments containing more than 70% (score 3) TrkA positive cells. Similar patchy expression was found in sections B and C, where number of positive cells reached mostly a maximum of score 2 in these areas. In section D a reduction of positive cells was observed with a maximal score of 2 in all parts of the nasal turbinates. In localization 5 only few TrkA-positive cells were present (**Figure 7D**).

The statistical evaluation started with a three-way ANOVA (I) for the expression of OMP, DCX, PCNA and TrkA including repeated measures for section, localization and respective marker. This was done to assess whether one marker was predominantly expressed in a certain area of the equine nose. Globally, significant differences between the sections and the localizations (*p* < 0.0001) as well as between the markers (*p* = 0.0037) were found. Additionally, the interaction between section and localization (*p* < 0.0010), between section and marker (*p* = 0.0005) and between localization and marker (*p* < 0.0001) was significant. This was also the case for the triple interaction between section, localization and marker (*p* = 0.0027). Accordingly, differences in presence of certain cell types depended on respective section and localization. Thus, the cell specific markers were expressed diversely within the equine nose. In general, number of cells expressing its specific protein was highest for the nasal turbinates with a significant reduction in rostral direction.

**FIGURE 8:**
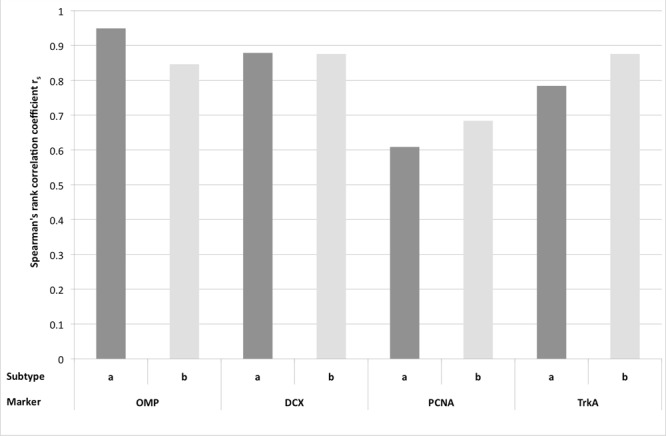
**Spearman’s rank correlation coefficient *r*_s_ for the expression of the immunohistological markers OMP, DCX, PCNA, and TrkA for the subtypes a and b of the olfactory epithelium**.

As a second approach (step II), the Spearman’s rank correlation (*r*_s)_ was calculated (**Figure 8**). The highest correlation was found for OMP and OE **type a** (*r*_s_ = 0.949). For OE **type b** and OMP correlation was still high, even though the value was slightly lower (0.846). Thus, expression of OMP strongly correlated with presence of OE **type a** and therefore, more adult OMP positive cells were detected in OE **type a** than in **type b**. DCX expression correlated also strongly with t**ype a** (0.879) but also with **type b** (0.876). For PCNA expression *r*_s_ was 0.609 for **type a**, still representing a distinct correlation. In contrast to OMP and DCX expression, *r*_s_ of PCNA positive cells was higher (0.684) for OE **type b**, pointing toward a stronger proliferation rate in the epithelium **type b**. Correlation with OE **type b** was even stronger for TrkA expression (0.876) than for type a (0.784) indicating more horizontal basal cells as progenitors in this OE subtype.

In summary, in OE **type a** more OMP and DCX positive cells were present and thus, more adult and juvenile neurons were present in OE **type a** than in OE **type b**. Vice versa, more PCNA and TrkA-positive cells were found in **type b** and according to this, more progenitor cells, proliferating and horizontal basal cells, were detected in OE **type b**.

## Discussion

Due to the unique features of the OE with direct exposure to the environment it is accessible for a broad range of insults, including neurotropic virus infections. For horses, different viruses, e.g., Borna disease virus, equine herpesvirus 1 (EHV-1), hendra virus, influenza virus, rabies virus, VSV can use this route. However, a detailed characterization of the equine OE was yet lacking. Studies to characterize the OE in domestic animals are rare (Deckner et al., 1993; Bock et al., 2009; Barrios et al., 2014a,b) or mainly focus on other components of the olfactory system like the olfactory bulb or the vomeronasal organ (Choi et al., 2010; Salazar and Sanchez-Quinteiro, 2011).

Therefore, in the present study, location within the nose, maturity, cytoarchitecture, and expression profile on a cellular level of the equine OE was characterized in detail and compared to existing data of canine and rodent OE. This could be helpful to address species-specific or species-conserved features in a comparative approach and thereby possibly also species-specific susceptibility to intranasal insults such as virus infections. Moreover, feasibility of rodent models by specifying conserved and contradictory characteristics could become more obvious.

### Localization of the Olfactory Epithelium in the Equine Nose

In horses the OE was located predominantly in the area of all three nasal turbinates at the most caudal parts of the nasal cavity but could reach the rostral end of the nasal turbinates. No dependence on age was detected regarding the intranasal localization of the OE. In a previous study in horses it was reported that the rostral endings of the nasal turbinates were covered exclusively with respiratory epithelium and OE was only detected on the nasal turbinates nearby the cribriform plate (Kumar et al., 2000). These discrepancies could be due to different sampling techniques since Kumar et al. (2000) only examined the caudal parts and the rostral endings of the nasal turbinates in contrast to our study where we investigated the entire nasal turbinates serially. In the canine nose the OE was found in the caudally located 6–7 cm of the nasal cavity (Bock et al., 2009) whereas in the horse it was detected in the caudal 6–8 cm in the equine nose. Thus, in dogs, it engages a proportionately bigger area within the nose, maybe reflecting the enormous smelling capability of canines (revied by Kavoi et al., 2010). Nickel et al. (2004) found canine OE also in some lateral areas at the basement of the turbinates. Furthermore, Kavoi et al. (2010) reported that the canine OE is more structured than the OE of herbivores concerning percentage of neurons, diameter of nerve fibers and number of cilia on the dendritic knobs. In rats, the OE is located at the caudal third of the nasal cavity, comprising the posterior regions of the nasal turbinates, roof and to a variable extent also of the nasal septum (Astic and Saucier, 1986; Strotmann et al., 1994a,b; Camara et al., 2006). This is comparable to the localization of the equine OE found in the present study. In humans, it was reported that the location of the OE is variable depending on age and damage by environmental insults (Patel and Pinto, 2014). However, the biggest amount of OE can be found at the cribriform plate, nasal roof, superior turbinate and in lower areas at the middle turbinate, posterior and middle nasal septum (Kern, 2000; Patel and Pinto, 2014) In the present study, the transition between olfactory and respiratory epithelium was fluent since regions of OE alternated with regions of respiratory epithelium in all areas examined. This has also been reported for the dog (Bock et al., 2009). Summarizing, the main proportion of the equine OE was found on the three nasal turbinates with a significant reduction in rostral direction. This has not been described in other species to date. Approximately from the rostral end of the mid nasal turbinate only respiratory epithelium was detected.

### Distribution of the Subtypes of the Equine OE

Interestingly, the OE is subject to ongoing regeneration with active and inactive zones next to each other (Graziadei and Graziadei, 1979). Bock et al. (2009) reported two different subtypes of the canine OE on the base of histology and cell specific marker expression. The designated **type A** represented the more mature subtype with large, round neurons while **type B** was thought to be the more immature type with the smaller, oval-shaped juvenile neurons (Bock et al., 2009). This has so far not been reported for the OE of rats and humans. Interestingly, in horses, also two distinct subtypes of OE were found morphologically and by percentage of specific cell types within the respective subtype. However, varying staining patterns for OMP and DCX in the OE subtypes as described for the dog were not that pronounced in the equine OE. Equine OE **type a** was considerably comparable to the canine OE **type A** while in equine OE **type b** less mature ORNs were present leading to a more bulky appearance. However, the number of juvenile ORNs was virtually similar to the canine OE type B (Bock et al., 2009). In dogs, cilia and terminal knobs were completely absent in the OE type B (Bock et al., 2009). In horses, these structures were still present but reduced in number resulting in a smoother appearance of the epithelial surface. The subtypes were furthermore differentiated by the location of the nuclei of the ORNs in relation to the epithelial surface and the thickness of the epithelium. In the equine OE the thickness of **type b** was reduced in contrast to the thickness of type B in the canine OE. In dogs, the more mature OE (type A) was located close to the cribriform plate (1 cm), while the OE type B occupied only the more rostrally located regions. This tendency could be confirmed for the equine OE since a predominance of **type b** in the mid and ventral parts of the nasal turbinates from section C onward was detected and was paralleled to reduction of **type a**.

The observed differences between equine and canine subtypes could base either on species-specific factors or on the age of the animals investigated. Bock et al. (2009) analyzed the OE of younger dogs with possibly more proliferative activity whereas the age of the horses investigated in the present study ranged from 3 to 21 years. However, in our initial age-spanning study no significant differences were found regarding the presence of OE subtypes. In another study, no subtypes of canine OE were found, neither in fetuses and neonates nor in adult individuals (Barrios et al., 2014a). However, these data based only on morphology without any further immunohistochemical analysis. Moreover, studies by Bock et al. (2009) and Barrios et al. (2014a) differed furthermore in sampling techniques.

Thus, in horses and dogs subtypes of the OE exist with remarkably overlapping characteristics regarding morphology, location, and decreasing amount in rostral direction. For horses OE subtypes are described for the first time. For the rat OE no subtypes have been described so far. However, the rodent OE resembles more the canine and equine OE type A/**a**, respectively.

### Cellular Protein Expression Profile of the Equine OE

The cellular composition of the equine OE was analyzed by cell specific proteins with conserved expression in rats, mice, human and dogs (Buiakova et al., 1994; Weiler and Benali, 2005; Murdoch and Roskams, 2007; Bock et al., 2009; Barrios et al., 2014a). This allowed to identify mature and juvenile ORNs as well as their progenitors, the horizontal and proliferating basal cells.

Olfactory marker protein as marker for adult olfactory neurons plays an important role during signal detection and transduction during smelling (Buiakova et al., 1996; Reisert et al., 2007). In horses strong OMP expression correlated significantly with morphologically identified presence of OE. Regions with scant OMP expression as described for dogs (Bock et al., 2009) were not detected in the equine OE. Whether this might be an age-related effect only present in younger dogs or might be due to a generally higher occurrence of immature OE in dogs due to higher proliferation rate needed for extensive olfactory properties has to be further investigated. OMP was found in the cytoplasm and nuclei in the equine OE as previously described (Farbman and Margolis, 1980; Koo et al., 2004; Bock et al., 2009). However, for rats and mice, a predominance of cytoplasmatic staining has also been reported (Buiakova et al., 1994, 1996; Weiler and Benali, 2005). In dogs, nuclear staining was mainly found in canine OE type A and the cytoplasmic staining dominated in type B so that Bock et al. (2009) discussed a functional change of OMP in the OE subtypes. Similar tendencies for a more pronounced staining of the cytoplasm were also present in the equine **type b**. Furthermore, in horses, OE **type a** contained a higher number of mature ORNs than **type b** and OE **type a** was composed of more cell rows. A significant reduction of OMP positive cells was observed in caudorostral direction similarly as for the canine OE (Bock et al., 2009). This indicates a higher proportion of mature ORNs in the caudal areas and a reduced differentiation into mature and functional neurons in the more rostrally located regions of the OE in horses and in dogs. Whether this represents an effect caused by various insults which can reach the rostral regions more easily or is due to an intrinsically higher proliferation rate as protection against incoming insults yet remains speculative. For mice it is known that OMP is first expressed when the neurons get connected to the olfactory bulb, thus, when olfactory signals and potentially also harmful agents can be transmitted to the CNS (Graziadei et al., 1980).

In several species DCX has been proved as reliable marker for juvenile neurons in which this protein binds to the microtubuli (Brown et al., 2003; Carson et al., 2006; Murdoch and Roskams, 2007). In horses, no differences concerning the intracellular expression pattern of DCX between **types a** and **b** were noted. Moreover, in the equine OE the DCX staining pattern was comparable to the rat OE (own unpublished data). A reduced expression was seen for the nasal septum from sections A to B and for the nasal turbinates not before section D. A decreasing amount of DCX expression in rostral direction has not been reported for the canine or rodent OE so far. Bock et al. (2009) did also not observe a reduction of TrkB-positive juvenile neurons in rostral direction. These finding suggest that in the most caudally located sections of the equine OE DCX was expressed to a similar extent. This might indicate a constant renewal albeit on lower level of the OE in horses in **type a**. The reduced DCX expression in section D correlated with a lower number of OMP positive ORNs in the rostral olfactory area because juvenile neurons are the progenitors of the mature ORNs. This reduced differentiation into mature neurons might act as precaution against incoming toxic insults and infections.

Proliferating cell nuclear antigen expression was used to identify the proliferating basal cells. Moreover, Ki-67 expression is widely used for the characterization of the humans, rodents and carnivore OE and both markers are useful to detect dividing cells (Carter et al., 2004; Murdoch and Roskams, 2007; Bock et al., 2009; Holbrook et al., 2011). PCNA expression has already been described for the equine sensory epithelium of the vomeronasal organ (Miller et al., 2003) and was also found in the rat OE (own unpublished data). Typically, PCNA positive cells were found in small groups indicating that proliferating areas intermingle with non-proliferating regions (Graziadei and Graziadei, 1979). While in the most caudally located sections of the nasal turbinates the number of PCNA positive cells was nearly constant only few positive cells were found at the caudal nasal septum. PCNA expression decreased significantly in rostral direction, thereby corresponding to the reduced expression of OMP and DCX. Thus, a higher proliferative activity in rostral areas as described for the dog by Ki-67 expression (Bock et al., 2009) was not confirmed for the equine OE. Thus, lacking differentiation into mature olfactory neurons or a higher apoptotic index might rather play a protective role in the equine OE against incoming insults. However, it should be mentioned that in the present study only horses without any neurological or respiratory disease were included so that this aspect might be better investigated in animals affected by a respective disease.

Horizontal basal cells of equine OE were identified by TrkA, the high-affinity receptor for the NGF (Feron et al., 2008). In the present study, TrkA staining pattern in the equine OE was similar as described for the equine vomeronasal organ (Garcia-Suarez et al., 1997). In horses, TrkA positive cells were found in almost constant number in caudal areas with a significantly reduced presence of positive cells in rostral direction. Neither for dogs nor for rats comparable analyses are available till date.

Presence of specific cell types in one of the OE subtypes was confirmed statistically by the Spearman’s rank correlation coefficient *r*_s_. OMP and DCX expression revealed the strongest correlation to OE **type a**, indicating a pronounced expression in this subtype of the equine OE. Vice versa, PCNA and TrkA expression were stronger correlated to OE **type b**. This substantiated to the suggestion by Bock et al. (2009) that in the equine OE **type b** also represents the more immature OE with more cellular precursors in comparison to the higher expression of proteins representative for mature and juvenile ORNs in OE **type a**.

### Comprehensive Discussion across Species

Altogether, the equine OE resembles the OE of other mammals, amongst them rodents and dogs, as well as humans regarding location within the nose, cytoarchitecture turnover, proliferation and cellular protein expression. However, the total area of OE is remarkably higher in dogs and could reflect the enormous smelling capacity of this species. The pronounced caudal location of mature ORNs might serve as protection against incoming substances and infectious agents using the olfactory pathway to the brain. Turnover rate of OE seem to be species-specific especially in rostral areas and might reflect species-specific requirements to keep the epithelium functional. Cytoarchitecture of OE subtypes might underlie age-related factors but this was not detected in the equine cohort investigated. The OE is composed of several cell types with different characteristics and function and these cell types are present in different amounts depending on localization and section of the OE within the nose. Whether this fact might influence susceptibility for certain insults such as viral infections warrants further investigation. However, if mature ORNs which are the main target for most of neurotropic viruses are more abundant in one species *per se* or if these cells are accessible more easily due to presence in more rostral locations it cannot be ruled out that this might enhance the chance to get infected. For horses, BoDV-1 is supposed to reach the CNS via the olfactory pathway which was confirmed in the rat model. Since the rat OE is remarkably comparable to the equine OE **type a** this seems feasible. In contrast, large areas of the canine OE belong to OE type B (Bock et al., 2009). Since dogs are in general resistant to infection with BoDV-1 one might suggest that the limited amount of OE type A (our **type a**) present only in very caudal regions of the nasal cavity might play a role. In contrast, in horses, the main dead end host of BoDV-1, large areas of the OE belong to the more mature **type a**. Since the olfactory receptor(s) of BoDV-1 are yet unknown, conclusions on the role of species-specific receptors are not feasible. In summary, the OE comprises species-conserved features regarding general cellular composition, cytoarchitecture and protein expression profile but also species-specific characteristics regarding localization within the nasal cavity and presence of epithelial subtypes. Whether this might contribute to specific susceptibilities to certain intranasal insults hast to be investigated in further studies.

## Author Contributions

AK: designed the study, carried out and evaluated the histology, discussed the statistical analysis, wrote the manuscript; SW: designed the study, discussed the data, revised the manuscript; KF: designed the study, performed statistical analysis, evaluated the statistical data, revised the manuscript; CH: designed the study, evaluated the histology, discussed the statistical analysis, wrote and revised the manuscript.

## Conflict of Interest Statement

The authors declare that the research was conducted in the absence of any commercial or financial relationships that could be construed as a potential conflict of interest.
