# Supplementary material for: Intranasal Location and Immunohistochemical Characterization of the Equine Olfactory Epithelium
Source: Front Neuroanat. 2016 Oct 13;10:97. doi: 10.3389/fnana.2016.00097 (PMC5061740; doi:10.3389/fnana.2016.00097)
Supplement: Supplementary file 1 [file Table_1.PDF]

Table S1: Overview of the statistical analysis. Analysis was carried out in an exploratory manner in different steps since neither the conditions for the application for parametric nor for non-parametric statistical procedures were given exactly.

**Percentage of the OE in the equine nose**	
**(I)**	**Two-way ANOVA:** relationship between percentage of OE and section or localization; interaction between factors
**(II)**	**Exact Friedman test:** interaction between localization and section
**(III)**	**Exact Friedman test:** comparison of localizations section-wise
**(IV)**	**Exact Friedman test:** comparison of the sections localization-wise
**Subtypes of the OE in the equine nose**	
**(I)**	**Three-way ANOVA:** relationship between percentages of subtypes of OE and section or localization; dual or triple interactions between factors
**(II)**	**Exact Friedman test:**<br>• interaction between localization and section for the subtypes of the OE<br>• interaction between sections and subtypes of the OE for the localizations<br>• interaction between localizations and subtypes of the OE
**(III)**	**Exact Friedman test:**<br>• comparison of localizations section-wise<br>• comparison of subtypes of the OE section-wise<br>• comparison of subtypes of OE localization-wise
**(IV)**	**Exact Friedman test:**<br>• comparison of sections localization-wise<br>• comparison of sections subtype-wise<br>• comparison of localizations subtype-wise
**(V)**	**Exact Friedman test:** if interaction is absent<br>• comparison of localizations for mean of sections and vice versa<br>• comparison of sections for mean of subtypes and vice versa<br>• comparison of localizations for mean of subtypes and vice versa
**Immunohistochemical marker expression**	
**(I)**	**Three-way ANOVA:** relationship between marker expression and section or localization; dual or triple interactions between factors
**(II)**	**Spearman's rank correlation:** correlation of marker expression and subtype of the OE
