# Supplementary material for: Intranasal Location and Immunohistochemical Characterization of the Equine Olfactory Epithelium
Source: Front Neuroanat. 2016 Oct 13;10:97. doi: 10.3389/fnana.2016.00097 (PMC5061740; doi:10.3389/fnana.2016.00097)
Supplement: Supplementary file 2 [file Table_2.pdf]

Table S2a: Sections A - M split up for localizations 1-5; olfactory epithelium only detectable in A-D; localization 1: nasal septum, 2: dorsal part of the nasal turbinate, 3: mid part of the nasal turbinate, 4: ventral part of the nasal turbinate, 5: outer lining of the nasal cavity; mean: arithmetic mean of percentages of the OE of the 5 horses; SD: standard deviation, median: median of percentages of the OE

Section	Localization	Mean	SD	Median
A	1	60	34.6	80
	2	80	14.1	80
	3	80	14.1	80
	4	72	17.9	80
	5	0	0.0	0
B	1	4	8.9	0
	2	68	17.9	80
	3	64	8.9	60
	4	56	8.9	60
	5	0	0.0	0
C	1	0	0.0	0
	2	32	26.8	20
	3	36	26.1	40
	4	40	24.5	40
	5	2	4.5	0
D	1	0	0.0	0
	2	8	17.9	0
	3	12	11.0	20
	4	8	17.9	0
	5	0	0.0	0

Table S2b: Sections A - M split up for localizations 1-5 and types a and b; olfactory epithelium only detectable in A-D; localization 1: nasal septum, 2: dorsal part of the nasal turbinate, 3: mid part of the nasal turbinate, 4: ventral part of the nasal turbinate, 5: outer lining of the nasal cavity; mean: arithmetic mean of th percentage of OE of the 5 horses; SD: standard deviation; median: median of percentages of the OE

Section	Localization	Mean<br>Type a / Type b	SD<br>Type a / Type b	Median<br>Type a / Type b
A	1	44 / 16	28.8 / 11.4	60 / 20
	2	60 / 20	14.1 / 0	60 / 20
	3	58 / 22	14.8 / 4.5	60 / 20
	4	50 / 22	17.3 / 4.5	60 / 20
	5	0 / 0	0 / 0	0 / 0
B	1	2 / 2	4.5 / 4.5	0 / 0
	2	44 / 24	16.7 / 8.9	40 / 20
	3	42 / 22	13 / 8.4	40 / 20
	4	30 / 26	7.1 / 5.5	30 / 30
	5	0 / 0	0 / 0	0 / 0
C	1	0 / 0	0 / 0	0 / 0
	2	20 / 12	20 / 11	10 / 10
	3	14 / 22	15.2 / 13	10 / 30
	4	18 / 22	14.8 / 11	20 / 20
	5	0 / 2	0 / 4.5	0 / 0
D	1	0 / 0	0 / 0	0 / 0
	2	2 / 6	4.5 / 13.4	0 / 0
	3	4 / 8	5.5 / 8.4	0 / 10
	4	2 / 6	4.5 / 13.4	0 / 0
	5	0 / 0	0 / 0	0 / 0
