# Supplementary material for: Intranasal Location and Immunohistochemical Characterization of the Equine Olfactory Epithelium
Source: Front Neuroanat. 2016 Oct 13;10:97. doi: 10.3389/fnana.2016.00097 (PMC5061740; doi:10.3389/fnana.2016.00097)
Supplement: Supplementary file 3 [file Table_3.PDF]

Table S3a: Exact Friedman test, comparison of section and localizations of the equine OE; localization 1: nasal septum, 2: dorsal part of the nasal turbinate, 3: mid part of the nasal turbinate, 4: ventral part of the nasal turbinate, 5: outer lining of the nasal cavity; α: Bonferroni-Holm α-adjustment; dns: data not sufficient; *statistically significant

**Comparison of sections and localizations**		
**II Interaction between localization and section**		
Difference	p	α
B-A	0.1192	0.050
C-A	*0.0027	0.017
D-A	*0.0165	0.025
**III Comparison of localizations, split up for sections (interaction present)**		
Section	p	α
A	*0.0008	0.025
B	*<0.0001	0.013
C	*0.0004	0.017
D	0.22	0.050
**IV Comparison of sections, split up for localizations (interaction present)**		
Localization	p	α
1	*0.0156	0.025
2	*0.0001	0.010
3	*0.0001	0.013
4	*0.015	0.017
5	dns	0.050

Table S3b: Exact Friedman test, comparison of sections and localizations, split up for olfactory epithelium types a and b; localization 1: nasal septum, 2: dorsal part of the nasal turbinate, 3: mid part of the nasal turbinate, 4: ventral part of the nasal turbinate, 5: outer lining of the nasal cavity; α: Bonferroni-Holm α-adjustment; dns: data not sufficient; *statistically significant

**Comparison of sections and localizations, split up for types of the olfactory epithelium**					
Type a			***Type b***		
**II Interaction between section and localization**			**II Interaction between section and localization**		
Difference	p	α	Difference	p	α
B-A	0.0794	0.050	B-A	0.0739	0.050
C-A	*0.0033	0.017	C-A	0.1224	0.017
D-A	*0.0057	0.025	D-A	0.1738	0.025
**III Comparison of localizations, split up for sections (interaction present)**			**V Comparison of localizations for mean of sections (interaction absent)**		
Section	p	α	Section	p	α
A	*0.0019	0.017	Sections A-D	*<0.0001	0.050
B	*<0.0001	0.013			
C	*0.007	0.025			
D	0.76	0.050			
**IV Comparison of sections, split up for localizations (interaction present)**			**V Comparison of sections for mean of localizations (interaction absent)**		
Localization	p	α	Localization	p	α
1	*0.0156	0.025	Localizations 1-5	*0.0076	0.050
2	*<0.0001	0.010			
3	*<0.0001	0.013			
4	*0.0025	0.017			
5	dns	0.050			

Table S3c: Exact Friedman test, comparison of sections and types of olfactory epithelium, split up for localizations; localization 1: nasal septum, 2: dorsal part of the nasal turbinate, 3: mid part of the nasal turbinate, 4: ventral part of the nasal turbinate, 5: outer lining of the nasal cavity; Dif: difference; α: Bonferroni-Holm α-adjustment; dns: data not sufficient; *statistically significant

**Comparison of types of olfactory epithelium and sections, split up for localizations**														
Localization 1			***Localization 2***			***Localization 3***			***Localization 4***			***Localization 5***		
**II Interaction between type and section**			**II Interaction between type and section**			**II Interaction between type and section**			**II Interaction between type and section**			**II Interaction between type and section**		
Dif	p	α	Dif	p	α	Dif	p	α	Dif	p	α	Dif	p	α
types-sections	0.0625	0.050	types-sections	*0.0009	0.050	types-sections	*0.0004	0.050	types-sections	*0.0026	0.050	types-sections	dns	0.050
**V Comparison of types for mean of sections (interaction absent)**			**III Comparison of types, split up for sections (interaction present)**			**III Comparison of types, split up for sections (interaction present)**			**III Comparison of types, split up for sections (interaction present)**			**V Comparison of types for mean of sections (interaction absent)**		
Section	p	α	Section	p	α	Section	p	α	Section	p	α	Section	p	α
A-D	0.25	0.050	A	0.0625	0.013	A	0.0625	0.013	A	0.125	0.013	A-D	dns	0.05
			B	0.25	0.017	B	0.25	0.017	B	dns	0.017			
			C	dns	0.025	C	0.5	0.025	C	dns	0.025			
			D	dns	0.050	D	dns	0.050	D	dns	0.050			
**V Comparison of sections for mean of types (interaction absent)**			**IV Comparison of sections, split up for types (interaction present)**			**IV Comparison of sections, split up for types (interaction present)**			**IV Comparison of sections, split up for types (interaction present)**			**V Comparison of sections for mean of types (interaction absent)**		
Type	p	α	Type	p	α	Type	p	α	Type	p	α	Type	p	α
a/b	*0.0156	0.050	a	*<0.0001	0.025	a	*<0.0001	0.025	a	*0.0025	0.025	a/b	dns	0.05
			b	0.1468	0.050	b	0.1126	0.050	b	0.1094	0.050			

Table S3d: Exact Friedman test, comparison of localizations and types of olfactory epithelium, split up for sections; localization 1: nasal septum, 2: dorsal part of the nasal turbinate, 3: mid part of the nasal turbinate, 4: ventral part of the nasal turbinate, 5: outer lining of the nasal cavity; Dif: difference; α: Bonferroni-Holm α-adjustment; dns: data not sufficient; *statistically significant

**Comparison of localizations and type of olfactory epithelium, split up for sections**											
Section A			***Section B***			***Section C***			***Section D***		
**II Interaction between type and localization**			**II Interaction between type and localization**			**II Interaction between type and localization**			**II Interaction between type and localization**		
Dif	p	α	Dif	p	α	Dif	p	α	Dif	p	α
types-localizations	*0.0054	0.050	types-localizations	*0.024	0.050	types-localizations	0.18	0.050	types-localizations	1	0.050
**III Comparison of types, split up for localizations (interaction present)**			**III Comparison of types, split up for localizations (interaction present)**			**V Comparison of types for mean of localizations (interaction absent)**			**V Comparison of types for mean of localizations (interaction absent)**		
Localization	p	α	Localization	p	α	Localization	p	α	Localization	p	α
1	0.25	0.025	1	dns	0.025	1-5	1	0.050	1-5	0.5	0.050
2	0.0625	0.010	2	0.25	0.010						
3	0.0625	0.013	3	0.25	0.013						
4	0.125	0.017	4	dns	0.017						
5	dns	0.050	5	dns	0.050						
**IV Comparison of localizations, split up for types (interaction present)**			**IV Comparison of localizations, split up for types (interaction present)**			**V Comparison of localizations for mean of types (interaction absent)**			**V Comparison of localizations for mean of types (interaction absent)**		
Type	p	α	Type	p	α	Type	p	α	Type	p	α
a	*0.0019	0.025	a	*<0.0001	0.025	a/b	0.0004	0.050	a/b	0.22	0.050
b	*0.0034	0.050	b	*0.0001	0.050						
